# How well do general practitioners know their elderly patients’ social relations and feelings of loneliness?

**DOI:** 10.1186/s12875-018-0721-x

**Published:** 2018-02-26

**Authors:** Tina Drud Due, Håkon Sandholdt, Volkert Dirk Siersma, Frans Boch Waldorff

**Affiliations:** 1 Research Unit for General Practice and Section of General Practice, Department of Public Health, University of Copenhagen, Copenhagen, Denmark; 2 Research Unit of General Practice, Department of Public Health, University of Southern Denmark, Odense, Denmark

**Keywords:** Social relations, Loneliness, General practice, Primary care

## Abstract

**Background:**

Social relationships are important to people and affect their quality of life, morbidity and mortality. The aim of this study was to examine the correlation between elderly patients’ descriptions of their social relations and feelings of loneliness, and their general practitioners’ assessments of these.

**Methods:**

Cross-sectional study in 12 general practices in the Capital Region of Denmark. During a three-week period each practice asked their patients aged 65 and older to fill out a questionnaire regarding health, social relations and loneliness; the general practitioner (GP) filled out a matching questionnaire regarding their perception of the patient’s social relations and loneliness. Data were collected from February to September 2014.

**Results:**

Of the 767 eligible patients 476 were included in the study. For 447 patients both GP and patient had answered at least one question on loneliness or social participation. The correlations between patients’ and GPs’ answers regarding social participation and loneliness were low (0.04–0.26). While GPs were less able to identify lonely patients and patients with low social participation, they were better at identifying not-lonely patients or those with high social participation. It was especially difficult for GPs to identify lonely patients when they were not living alone or if the GP believed the patient had high social participation.

**Conclusion:**

GPs have difficulty identifying patients who are lonely or have low social participation and this ability is further diminished when the patients do not live alone or if the GP believes them to have high social participation. Given the consequences of loneliness and limited social participation on patients’ health and well-being, and GPs’ limited ability to identify these patients, GPs’ obligations and resources in this area need to be clarified.

**Electronic supplementary material:**

The online version of this article (10.1186/s12875-018-0721-x) contains supplementary material, which is available to authorized users.

## Background

In studies among elderly people in Europe, the prevalence of chronic or frequent loneliness varies and may be as high as 45% depending on definitions, measurements, population sample and age groups [1–4]. Social relations and feelings of loneliness affect quality of life, management of chronic diseases, morbidity and mortality [5–9]. This effect is similar to other known risk factors such as smoking, alcohol, physical inactivity and hypertension [10]. In addition to the effects of social relations on health, there is also a tendency towards reverse causality. Various physical disabilities can lead to reduced mobility, which can make it difficult to participate in social activities [11–13].

According to the WONCA (World Organization of Family Doctors) definition, general practitioners (GPs) are personal doctors, primarily responsible for the provision of comprehensive and continuing care to individuals seeking medical care in the context of their family, community, and culture. Furthermore, GPs integrate physical, psychological, social, cultural and existential factors, utilizing the knowledge and trust engendered by repeated contacts [14]. Due to the adverse health effects of loneliness, it may be important for GPs to identify lonely elderly patients. However, there is very sparse knowledge of how GPs handle patients with loneliness and whether GPs are able to identify them accurately [4, 15, 16].

The aim of this study was to examine the correlation between elderly patients’ descriptions of their social relations and feelings of loneliness with their GPs assessments thereof.

## Methods

### Healthcare setting

In Denmark, health care is mostly tax financed and includes free and direct access to GPs. Nearly 98% of all Danes are affiliated with a GP clinic, and GPs serve as gate-keepers in the health care system [17].

### Subjects

**Fig. 1:**
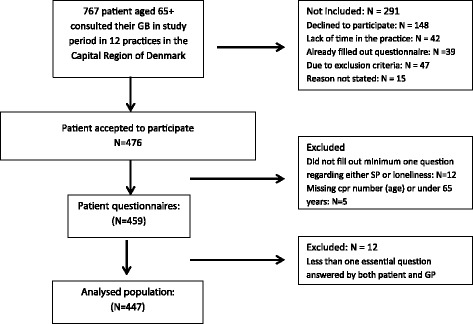
Flowchart

Twelve practices with a total of 20 GPs in the Copenhagen area (urban) and Bornholm (rural) participated in this study. In each practice, patient participants were recruited during a three-week period from February – September 2014. Patients aged 65 and older consulting their GP, regardless of the reason for their encounter, were asked to participate in the study. Patients unable to speak or read Danish, unable to answer the questionnaire, unable to sign an informed consent, or with severe acute or terminal illness were excluded (Fig. 1). All patients gave informed written consent for participation in the study. In the waiting room before the consultation, participating patients were asked to complete a patient questionnaire. If a patient had agreed to participate, the GP filled out a physician questionnaire about that patient before the patient entered the consultation room. The practice filled out a log of both participating and non-participating patients and noted reasons for non-participation. All GPs and their staff were instructed about the project by the first author. The GPs received a fee of €18 for each patient recruited in the project.

### Patient questionnaire

The questionnaire consisted of three parts, with a total of 20 questions. The questions were closed-ended with categorical and ordinal response categories:

1. The first part included sociodemographic questions, and questions about use of home care and patient affiliation to the practice.
2. The second part included questions about health, smoking and alcohol consumption:
  - Self-rated health was measured with a single item derived from the SF-36 health questionnaire *“In general, would you say your health is…”,* with five response categories: excellent, very good, good, fair and poor [18]. The Danish translation of the questionnaire has been rigorously tested [19].
  - Quality of life: The patients completed the Danish approved version of the EQ-5D questionnaire. EQ-5D measures five dimensions – mobility, self-care, usual activities, pain/discomfort, and anxiety/depression – each by three levels of severity; no problem, some or moderate problems, or extreme problems [20]**.**
  - Questions about mobility and ability to see/read a newspaper text and hear a normal conversation used items from the Danish National Health Interview Surveys with the following answer categories; able without difficulty, with a little difficulty, with a lot of difficulty, or unable to [21]
  - Questions about smoking and drinking habits were included using items from the Danish National Health Interview Surveys [21]. The question regarding drinking habits was simplified to use of alcohol per week instead of per each day in the last week.
3. The third part included information about social participation (SP) and feelings of loneliness. This section included:
  - Social participation: Social participation was measured by three questions: *“How often did you within the last month…”* (a) h*ave visitors at home?* (b) *visit others?* and (c) *participate in social activities outside home?* With the response categories *“at least once a week”*, *“less than once a week”,* or *“never”*. The three questions combined describe the participant’s level of social participation [6].
  - Loneliness was measured using the following item: *“Does it ever occur that you feel lonely?”*. With the response categories *“Yes - often”*, *“Yes - occasionally”*, *“Yes - but rarely”* and *“No”*. This question is from the Danish Longitudinal Study on Aging, which is a survey collecting data every five years among elderly people in Denmark [22]. We also used the following two questions from the Danish National Health Interview Surveys [21]: *“Does it happen that you are alone even though you wanted to be with others?”* and *“Do you have someone to talk to if you have problems or need support?”*.
  - One follow-up question was included for participants who stated they were lonely: a question regarding whether they had talked to their GP about loneliness.

Based on the three questions about social participation we computed two different social participation scores. We applied the previously used two-level social participation score by Avlund el al [6]. When summarizing the score across the three questions the answer, *“at least once a week”* is assigned 1 point in each question, while the other answers are assigned 0 points. A total of 3 points is considered high SP, while a total score of 0–2 is low SP. Hence, in this dichotomized score participants have to respond *“at least once a week”* in all three questions to have high social participation. However, we also constructed our own three-level social participation score, since in a previous study we found considerable differences in feelings of loneliness among people with what could be described as medium and low SP [23]. In our score, we assigned 1 point to the answer *“at least once a week”*, 2 points to the answer *“less than once a week”* and the 3 points to the answer *“never”*. Hence, this three-level social participation score yielded scores ranging from 3 to 9, with lower numbers representing higher social participation. We decided that high SP was 3 points equivalent to the scale by Avlund et al. (all three questions answered with *“at least once a week”*), medium SP was 4 and 5 points and low SP was 6 points or more.

The loneliness question was dichotomized, so the responses “Yes - often” and “Yes – occasionally” were labelled as “lonely” and the responses “Yes - but rarely” and “No” were labelled “not lonely”.

### Physician questionnaire

The GP questionnaire consisted of six questions corresponding directly to the questions on social participation and loneliness in the patient questionnaire, plus one question regarding the patient’s chronic diseases. The six questions corresponding to the patient questionnaire were rephrased. For example, the question “W*ithin the last month how often did you have visitors at home?”* was rephrased to *“Within the last month how often do you think your patient had visitors at home?”*

### Statistics

Patients were included in the analysis if at least one question about loneliness or social participation was answered by both patient and GP. Differences in background variables between participants and non-participants were assessed by chi-squared tests. Agreement between the responses from patients and GPs concerning social participation and loneliness was measured by an unadjusted Kappa statistic. To assess the GPs’ ability to identify patients who were lonely and not lonely, and to rate their patients’ level of social participation, we constructed the corresponding frequency tables and computed the sensitivity (the ability to identify the lonely patients or those with low SP) and specificity (the ability to identify the not-lonely or those with high SP) therein. An investigation into which variables were associated with agreement between patients and GPs regarding loneliness was completed separately for lonely patients and not-lonely patients, i.e. separate analyses into sensitivity and specificity, because different characteristics might influence the GPs ability to identify lonely patients and not-lonely patients. For the two loneliness questions from the Danish National Health Interview Surveys we calculated Kappa coefficients, but they were not included in the conditional distribution analysis, because we in a prior study found them highly associated with the loneliness question [23]. We used chi-squared tests, and used Monte Carlo simulated *P*-values if a cell count was five or less. A significance level of 0.05 was chosen and missing data were omitted from the analysis.

## Results

A total of 767 patients were eligible and 476 patients participated in the study (Fig. 1). Five patients were excluded due to missing social security number (inability to identify age) or because they were under 65 years of age. Included patients were significantly younger than non-included patients (41% and 57%, respectively, were over 75 years old) (*p* = 0.0002), while the gender distribution was equal (57% and 59% women) (*p* = 0.7751). In 447 individuals, we had the minimum of one matching question answered by both patient and GP. A total of 24 patients were missing from the analysis either because they had not responded to the loneliness question or one of the SP questions, or because their GP had not filled out the questionnaire. Of these, 71% were women and 21% were over 75 years old. Of the 12 patients missing only because the GP had not responded, 25% were lonely.

In the analysed population, the average age was 73.8 years and 57.4% were women. The majority (76.5%) suffered from chronic conditions, and 46.3% were living alone (Table 1). According to our definition, 18.2% had low social participation and 17.6% reported feeling chronically or frequently lonely.

**Table 1:** Distribution of patient characteristics

Characteristics		Total n (%)
Gender	Men	189 (42.3)
	Women	258 (57.4)
Age	65–75	265 (59.3)
	76+	182 (40.7)
Living alone	Yes	199 (46.3)
	No	231 (53.7)
Time associated with the GP	<= 5 years	53 (12.3)
	> 5 years	379 (87.7)
Self-rated health status	Excellent / Very good	132 (30.8)
	Good	207 (48.2)
	Less good / bad	90 (21.0)
Chronic disease	Yes	342 (76.5)
	No	105 (23.5)
Feeling anxious or depressed	No anxiety or depression	352 (82.1)
	Moderate/severe anxiety or depression	77 (17.9)
Visits by friends or family in the last month	At least once a week	299 (68.0)
	Less than once a week	127 (28.8)
	Never	14 (3.2)
Visited friends or family in the last month	At least once a week	257 (58.5)
	Less than once a week	170 (38.7)
	Never	12 (2.8)
Participated in leisure activities outside the home in the last month	At least once a week	267 (62.0)
	Less than once a week	103 (23.9)
	Never	61 (14.1)
Patient reported social participation	High	152 (35.8)
	Medium	195 (46.0)
	Low	77 (18.2)
Loneliness	Lonely	78 (17.6)
	Not lonely	365 (82.4)
Talked to GP if lonely	Yes	12 (15.2)
	No	67 (84.8)
Alone though wanting to be with others	Often/occasionally Rarely/no	88 (19.8) 356 (80,2)
Having someone to talk to if problems or needing support	Often/mostly sometimes/no	394 (88.7) 50 (11.3)

The correlations between the patients’ answers and the assessments by the GPs were quite low for all questions with kappa-values ranging from 0.04 to 0.26 (Table 2).

**Table 2:** Agreements between GPs’ and patients’ answers (Cohen’s kappa)

Question	Kappa coefficient
Visits by friends or family in the last month (three levels)	0.1782
Visited friends or family in the last month (three levels)	0.0821
Participated in leisure activities outside the home in the last month (three levels)	0.1575
Visits by friends or family in the last month (two levels: minimum weekly/less)	0.2088
Visited friends or family in the last month (two levels: minimum weekly/less)	0.1168
Participated in leisure activities outside the home in the last month(two levels: minimum weekly/less)	0.2348
Loneliness (four levels)	0.1801
Loneliness (two levels)	0.2607
Social participation (dichotomized score by Avlund et al.)	0.1576
Social participation (our three level score)	0.1355
Alone though wanting to be with others	0.1621
Having someone to talk to if problems or needing support	0.0358

As seen in Tables 3, 17.6% of the patients described that they often or occasionally felt lonely and the GPs assessed 23.2% of the patients to be lonely. However, they only identified 47.4% of the patients who had answered they were lonely (sensitivity), and the majority of the patients GPs assessed as lonely were not. Their ability to identify the not lonely patients (specificity) was 81.9%. Likewise, the GPs only identified a third of the patients that had a low social participation (sensitivity) (Table 4) and only 54.6% of the patients if combining low and medium in a dichotomized version of social participation. Again, the specificity was higher, since their ability to correctly identify those with high social participation was 62.8%. Only 15.2% of the patients that reported being lonely had *“often”* or *“sometimes”* discussed it with their general practitioner (Table 1).

**Table 3:** Agreement about patients’ feelings of loneliness

Patients’ response	Often/occasionally	Rarely/no	Total
GPs’ response			
Often/occasionally	37 (47.4)	66 (18.1)	103
Rarely/no	41 (52.6)	299 (81.9)	340
Total	78	365	443

**Table 4:** Agreement about patients’ social participation

Patients’ response	High	Medium	Low	Total
GPs’ response				
High	91 (62.8)	91 (48.4)	28 (37.8)	210
Medium	39 (29.9)	70 (37.2)	24 (32.4)	133
Low	15 (10.3)	27 (14.4)	22 (29.7)	64
Total	145	188	74	407

As seen in Table 5, GPs had more difficulty identifying the lonely patients if they were not living alone (*p* < 0.0001). However, even among patients living alone they only identified a little over half of the lonely patients. Likewise, they were better at identifying the not lonely patients among those not living alone (*p* < 0.0001). Furthermore, GPs’ ability to identify lonely and not lonely patients was associated with their rating of the patients’ social participation (*p* = 0.0004 for lonely patients and *p* < 0.0001 for not-lonely patients), while there was no difference in relation to the patient-reported social participation (*p* = 0.9789 and *p* = 0.7327) (neither the three individual SP questions nor the cumulative score). When GPs assessed their patients’ social participation to be lower, they were better at identifying lonely patients, and likewise when they assessed their patients’ social participation to be higher, they were also better at identifying non-lonely patients. While the patients’ self-rated health status was not associated with GPs’ ability to identify the lonely patients, a better self-rated health status was associated with an increase in the GPs’ ability to identify the non-lonely patients. The length of time the patient had been associated with the practice, the patients’ gender and age, chronic disease status and if the patients’ felt anxious or depressed had no influence on whether the GPs could distinguish between lonely and non-lonely patients.

**Table 5:** Test for variables influencing GPs ability to identify lonely and non-lonely patients

		Lonely patients			Not lonely patients		
		GP: Not lonely	GP: Lonely	*P*-value	GP: Not lonely	GP: Lonely	*P*-value
Gender	Men	14 (56.0)	11 (44.0)		134 (82.2)	29 (17.8)	
	Women	27 (50.9)	26 (49.1)	0.6764	165 (81.7)	37 (18.3)	0.8968
Age	65–75	22 (53.7)	19 (46.3)		188 (84.3)	35 (15.7)	
	76+	19 (51.4)	18 (48.7)	0.8385	111 (78.2)	31 (21.8)	0.1376
Living alone	Yes	21 (40.4)	31 (59.6)		94 (65.7)	49 (34.3)	
	No	19 (90.5)	2 (9.5)	<.0001*	193 (91.9)	17 (8.1)	<.0001
Time associated with the GP	<= 5 years	7 (58.3)	5 (41.7)		38 (92.7)	3 (7.3)	
	> 5 years	33 (54.1)	28 (45.9)	0.7876	253 (80.3)	62 (19.7)	0.0802*
Self-rated health status	Excellent / Very good	11 (73.3)	4 (26.7)		100 (87.0)	15 (13.0)	
	Good	16 (53.3)	14 (46.7)		144 (81.8)	32 (18.2)	
	Less good / bad	13 (48.2)	14 (51.8)	0.2831*	43 (69.4)	19 (30.6)	0.0160
Chronic disease	Yes	34 (53.1)	30 (46.9)		77 (85.6)	13 (14.4)	
	No	7 (50.0)	7 (50.0)	0.8320	222 (80.7)	53 (19.3)	0.3016
Feeling anxious or depressed	No anxiety or depression	20 (57.1)	15 (42.9)		257 (81.6)	58 (18.4)	
	Moderate/severe anxiety or depression	19 (52.8)	17 (47.2)	0.7117	33 (82.5)	7 (17.5)	0.8882
Visits by friends or family in the last month	At least once a week	20 (52.6)	18 (47.4)		215 (83.0)	44 (17.0)	
	Less than once a week	19 (55.9)	15 (44.1)		75 (81.5)	17 (18.5)	
	Never	2 (66.7)	1 (33.3)	0.9261*	7 (70.0)	3 (30.0)	0.6092*
Visited friends or family in the last month	At least once a week	17 (53.1)	15 (46.9)		186 (83.0)	38 (17.0)	
	Less than once a week	22 (53.7)	19 (46.3)		105 (82.7)	22 (17.3)	
	Never	2 (50.0)	2 (50.0)	1.000*	5 (62.5)	3 (37.5)	0.4974
Participated in leisure activities outside the home in the last month	At least once a week	21 (53.9)	18 (46.1)		190 (84.1)	36 (15.9)	
	Less than once a week	9 (50.0)	9 (50.0)		68 (80.0)	17 (20.0)	
	Never	10 (52.6)	9 (47.4)	0.9641	31 (75.6)	10 (24.4)	0.3628
GP assessed social participation	High	17 (73.9)	6 (26.1)		177 (91.7)	16 (8.3)	
	Medium	19 (67.9)	9 (32.1)		95 (84.8)	17 (15.2)	
	Low	5 (21.7)	18 (78.3)	0.0004	21 (44.7)	26 (55.3)	<.0001
Patient reported social participation	High	10 (52.6)	9 (47.4)		111 (84.1)	21 (15.9)	
	Medium	15 (55.6)	12 (44.4)		137 (82.0)	30 (18.0)	
	Low	15 (53.6)	13 (46.4)	0.9789	38 (79.2)	10 (20.8)	0.7327

*Monte Carlo simulated *P*-values

## Discussion

In this study, we found that the correlations between the patients’ and the GPs responses regarding social participation and loneliness were low. 17.6% of the elderly patients consulting their GP were frequently or chronically lonely. However, the GPs only identified about half of them and in fact incorrectly identified a fifth of their non-lonely patients as lonely. Likewise, they only identified a third of the patients who had low social participation. Hence, the sensitivity (the ability to identify lonely patients or those with low SP) was very low, while the specificity (the ability to identify not-lonely or those with high SP) was better. The GPs had even more difficulty identifying lonely patients, if they were not living alone or if the GPs thought that they had high social participation. This suggests that it is difficult for GPs to identify these patients, due to cultural assumptions about loneliness and social participation, living arrangements and other factors. This also highlights a need to disseminate the result from our previous study, where we found a significant association between patients’ level of social participation and feelings of loneliness, but also that several patients were lonely despite having high social participation or were not lonely despite low social participation [23]. Furthermore, it would have been expected that GPs more correctly identified loneliness among patients they had consulted for more than five years, the chronically ill, those with low self-rated health, or those with depression and anxiety, because GPs likely see such patients more often. However, these variables did not seem to influence the sensitivity or specificity of the GPs assessment of the patients’ loneliness. To our knowledge, no other studies have examined GPs’ knowledge about their patients’ social participation and feelings of loneliness in this way.

The lonely patients in our study reported that they rarely discussed these feelings with their GP. In relation to this finding, qualitative studies have found that GPs rarely ask their patients directly about loneliness, but rather ask them either indirectly or not at all [15, 16]. Furthermore, although GPs perceive identification of loneliness as relevant, they experience uncertainty concerning their role and obligations and experience lack of time, therapeutic options and knowledge of how to deal with lonely patients [4, 15, 16].

In light of the WONCA definition of general practice, our study indicates that GPs’ knowledge of social aspects among their elderly patients may be incomplete. GPs’ limited ability to identify these patients, combined with the consequences that loneliness and low social participation have on patients’ health and well-being, may challenge the overall WONCA definition, and emphasize a need for debate and clarification of GPs’ obligations, optimal role, and abilities in this area. As seen in qualitative studies, GPs feel frustrated and powerless talking to patients about feelings of loneliness, if they cannot offer them any help [4, 16]. Hence, if this is considered a GP obligation, they need to be better trained at how to identify and help lonely patients, the time spent on this task needs to be acknowledged at the administrative level, and there has to be relevant resources available for the GPs to refer lonely patients.

It is a strength of this study that it was conducted in a general practice setting with patients who were consulting their GP independently of the study and were asked to participate regardless of the reason for their visit. Hence, this reflects a real-life setting which increases the generalizability of the findings and the importance of addressing the issues identified. On the other hand, since only patients able to visit the practice and to answer the questionnaire were included, it does not reflect all patients in the general practice setting. Likewise, included patients were significantly younger than the non-included patients. Apart from this, missing data does not appear to be a problem due the distributions of known variables and a limited number of participating patients missing from the analysed population. It is a potential limitation that we did not perform a pilot study of the complete questionnaire. However, since the question has been used in prior Danish population surveys and the participants in our study were able to answer the questions, we consider it reliable. Concerning the studied population size of 12 practices, we had hoped for more participating practices, but they proved difficult to recruit. However, there is no reason to suspect that the findings are not generalizable, since participating practices were geographically spread and included both solo and partnership practices. However, further studies assessing the extent of the issue of GPs limited knowledge about patients’ social participation and loneliness would be preferable. Though more participating patients would have ensured a higher cell count in parts of the conditional distribution analysis, we believe we have reached saturation in our data, since a sufficient number was available. By asking the GPs to fill out the questionnaire prior to the consultation, we limited the possible bias that could have arisen if they had asked patients these questions directly before filling out the GP questionnaire, and thus we collected more accurate data on their knowledge of the patients. However, the fact that we lack information about how often the GPs see the participating patients or when they had their last consultation could be confounding variables, since GPs might have a more accurate assessment of patients seen regularly or recently. However, on average patients in Denmark, aged 65–79 years consult their GP 11 times a year, and 16 times a year for those above 80 years [24]. Hence, it is likely that the patients in this study also visit their GP regularly.

## Conclusion

GPs have difficuly identifying patients who are lonely or have low social participation, and this ability is further diminished when the patients do not live alone or if the GP believes them to have high social participation. Given the consequences of loneliness and limited social participation on patients’ health and well-being and GPs’ limited ability to identify these patients, GPs’ obligations and resources in this area need to be clarified and potentially improved.

## Additional files

Additional file 1: GP questionnaire. (PDF 111 kb)

Additional file 2: Patient questionnaire. (PDF 329 kb)

## Abbreviations

GP: General practitioner
SP: Social participation
