# Supplementary material for: How well do general practitioners know their elderly patients’ social relations and feelings of loneliness?
Source: BMC Fam Pract. 2018 Feb 26;19:34. doi: 10.1186/s12875-018-0721-x (PMC5828068; doi:10.1186/s12875-018-0721-x)
Supplement: Supplementary file 1 — GP questionnaire. (PDF 111 kb) [file 12875_2018_721_MOESM1_ESM.pdf]

Skema 5

Deltagernummer

Sociale relationer hos ældre patienter i almen praksis

Spørgeskema til patienter

FORSKNINGSENHEDEN
FOR ALMEN PRAKSIS
København

Personlige forhold

1. Hvor lang var Deres skoleuddannelse?

Under 7 års skolegang	☐
7-8 års skolegang	☐
9-10 års skolegang	☐
Studenter eksamen, HF, HH eller HTX	☐
Andet (herunder skolegang i udlandet)	☐

2. Bor du alene?

Ja	☐
Nej	☐

3. Hvad er Deres ægteskabelige status rent juridisk

Gift	☐
Registreret partnerskab	☐
Separeret	☐
Skilt	☐
Enke/enkemand	☐
Ugift	☐

4. Hvorledes er Deres nuværende boligforhold?

Bor i lejlighed (ejer-, andel eller lejerbolig)	☐
Bor i pensionistbolig eller kollektivbolig	☐
Bor i beskyttet bolig	☐
Bor i hus (ejer- eller lejerbolig)	☐
Bor på plejehjem	☐
Andet, opgiv arten:___________________________________	☐

5. Modtager du hjemmehjælp/hjemmesygeplejerske?

Nej	☐
Ja	☐
Hvis ja, hvor mange timer om ugen	☐

6. Hvor længe har De været tilknyttet denne lægepraksis

Under 1 år	☐
1-5 år	☐
Over 5 år	☐

Deltagernummer

Helbred og sundhedsvaner

7. Hvordan synes De deres helbred er alt i alt

Fremragende	☐
Vældig godt	☐
Godt	☐
Mindre godt	☐
Dårligt	☐

8. Angiv ved at sætte et X i én af kasserne i hver gruppe, hvilke udsagn, der bedst beskriver Deres helbredstilstand i dag.

Bevægelighed

Jeg har ingen problemer med at gå omkring	☐
Jeg har nogle problemer med at gå omkring	☐
Jeg er bundet til sengen	☐

Personlig pleje

Jeg har ingen problemer med min personlige pleje	☐
Jeg har nogle problemer med at vaske mig eller klæde mig på	☐
Jeg kan ikke vaske mig eller klæde mig på	☐

Sædvanlige aktiviteter (f.eks. arbejde, husarbejde, familie- eller fritidsaktiviteter)

Jeg har ingen problemer med at udføre mine sædvanlige aktiviteter	☐
Jeg har nogle problemer med at udføre mine sædvanlige aktiviteter	☐
Jeg kan ikke udføre mine sædvanlige aktiviteter	☐

Smerter/ubehag

Jeg har ingen smerter eller ubehag	☐
Jeg har moderate smerter eller ubehag	☐
Jeg har ekstreme smerter eller ubehag	☐

Angst/depression

Jeg er ikke ængstelig eller deprimeret	☐
Jeg er moderat ængstelig eller deprimeret	☐
Jeg er ekstremt ængstelig eller deprimeret	☐

Deres helbredstilstand i dag

For at hjælpe folk med at udtrykke, hvor god eller dårlig en helbredstilstand er, har vi tegnet en skala (næsten ligesom et termometer), hvor den bedste helbredstilstand du kan forestille dig er markeret med 100, og den værste helbredstilstand du kan forestille dig er markeret med 0.

Vi beder Dem angive på denne skala, hvor god eller dårlig De mener Deres egen helbredstilstand er i dag.

Angiv dette ved at tegne en streg fra kassen nedenfor til et hvilket som helst punkt på skalaen, der viser, hvor god eller dårlig deres helbredstilstand er i dag.

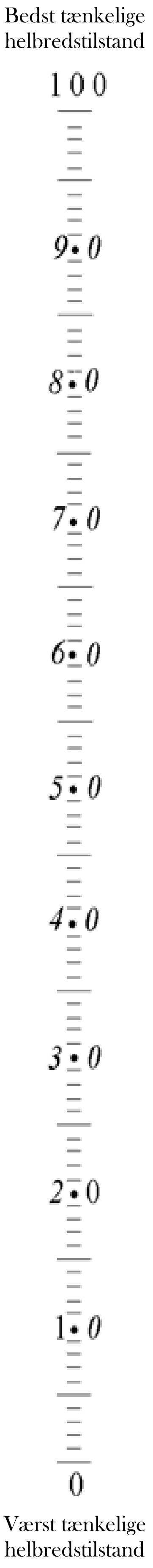

Deres helbredstilstand i dag

10. Hvorledes vil De bedømme Deres hukommelse?

Virkelig god	☐
God	☐
Nogenlunde	☐
Dårlig	☐
Meget dårlig	☐

11. Kan De normalt uden besvær, med lidt besvær, med meget besvær eller slet ikke gøre følgende

(Sæt ét X i hver linje)	Ja, uden besvær	Ja, med lidt besvær	Ja, med meget besvær	Nej slet ikke
Læse en almindelig avistekst (evt. med briller, hvis det normalt bruges)?	☐	☐	☐	☐
Høre, hvad der bliver sagt under en normal samtale mellem tre eller flere personer (evt. med høreapparat, hvis det normalt bruges)?	☐	☐	☐	☐
Gå 400 meter uden at hvile?	☐	☐	☐	☐
Gå op eller ned ad en trappe fra en etage til en anden uden at hvile?	☐	☐	☐	☐
Bære 5 kg (f.eks. indkøbsposer)?	☐	☐	☐	☐

12. Ryger De

Ja, hver dag	☐
Ja, mindst én gang om ugen	☐
Ja, sjældnere end hver uge	☐
Nej, jeg holdt op	☐
Nej, jeg aldrig røget	☐

13. Hvor meget af følgende har De i gennemsnit drukket om ugen de sidste 12 måneder?

	Skriv antal
Antal øl om ugen	
Antal glas vin om ugen (1 flaske =6 glas)	
Antal glas spiritus (2 cl.) om ugen	

Relationer med andre mennesker

14. Hvor ofte har De inden for den sidste måned haft besøg af venner, naboer eller familie i Deres hjem?

Mindst én gang om ugen	☐
Mindre end én gang om ugen	☐
Aldrig	☐
Ved ikke	☐

15. Hvor ofte har De inden for den sidste måned været på besøg i andre menneskers hjem?

Mindst én gang om ugen	☐
Mindre end én gang om ugen	☐
Aldrig	☐
Ved ikke	☐

16. Hvor ofte har De inden for den sidste måned deltaget i fritidsaktiviteter uden for Deres eget hjem (fx undervisning, foredrag, foreningsliv, klub, dagcenter, kirke, biograf, teater, museum, andet kulturelt)?

Mindst én gang om ugen	☐
Mindre end én gang om ugen	☐
Aldrig	☐
Ved ikke	☐

17. Sker det nogensinde, at De er alene, selvom De mest har lyst til at være sammen med andre?

Ja ofte	☐
Ja, en gang imellem	☐
Ja, men sjældent	☐
Nej	☐

18. Har De nogen at tale med, hvis De har problemer eller brug for støtte

Ja, ofte	☐
Ja, for det meste	☐
Ja, nogen gange	☐
Nej aldrig eller næsten aldrig	☐

19. Sker det nogensinde, at De føler dem ensom?

Ja ofte	☐
Ja, en gang imellem	☐
Ja, men sjældent	☐
Nej	☐

Hvis De ved spørgsmål 19 har svaret, at De ”Ja ofte” eller ”Ja, en gang imellem” føler Dem ensom, bedes De besvare det næste spørgsmål

20. Har De talt med Deres læge om, at De føler dem ensom

Ja	☐
Nej	☐

Tak for at De ville medvirke
