# Supplementary material for: How well do general practitioners know their elderly patients’ social relations and feelings of loneliness?
Source: BMC Fam Pract. 2018 Feb 26;19:34. doi: 10.1186/s12875-018-0721-x (PMC5828068; doi:10.1186/s12875-018-0721-x)
Supplement: Supplementary file 2 — Patient questionnaire. (PDF 329 kb) [file 12875_2018_721_MOESM2_ESM.pdf]

Skema 6

Deltagernummer

Sociale relationer hos ældre patienter i almen praksis

Spørgeskema til lægen

Udfyldes før konsultationen

FORSKNINGSENHEDEN FOR ALMEN PRAKSIS
København

1. **Hvor ofte tror du, at patienten inden for den sidste måned har haft besøg af venner, naboer eller familie i sit hjem?**

Mindst én gang om ugen	☐
Mindre end én gang om ugen	☐
Aldrig	☐
Ved ikke	☐

2. **Hvor ofte tror du, at patienten inden for den sidste måned har været på besøg i andre menneskers hjem?**

Mindst én gang om ugen	☐
Mindre end én gang om ugen	☐
Aldrig	☐
Ved ikke	☐

3. **Hvor ofte tror du, at patienten inden for den sidste måned har deltaget i fritidsaktiviteter uden for eget hjem (fx undervisning, foredrag, foreningsliv, klub, dagcenter, kirke, biograf, teater, museum, andet kulturelt)?**

Mindst én gang om ugen	☐
Mindre end én gang om ugen	☐
Aldrig	☐
Ved ikke	☐

4. **Tror du, at din patient nogensinde er alene, selvom han/hun mest har lyst til at være sammen med andre?**

Ja, ofte	☐
Ja, en gang imellem	☐
Ja, men sjældent	☐
Nej	☐

5. **Tror du, at din patient nogensinde føler sig ensom?**

Ja ofte	☐
Ja, en gang imellem	☐
Ja, men sjældent	☐
Nej	☐

6. Tror du, at din patient har nogen at tale med, hvis han/hun har problemer eller brug for støtte?

Ja, ofte	☐
Ja, for det meste	☐
Ja, nogen gange	☐
Nej aldrig eller næsten aldrig	☐

7. I tilfælde af, at din patient lider af kroniske sygdomme bedes disse venligst angives i listen nedenfor

**1.**
**2.**
**3.**
**4.**
**5.**
**6.**
**7.**
**8.**

Print eventuelt liste fra lægesystem. **HUSK at skrive deltagernummer på printet**
